# Success in the Dental Profession. No. 2

**Published:** 1862-06

**Authors:** C. C. Dills


### SUCCESS IN THE DENTAL PROFESSION.

BY C. C. DILLS, D. D. S.

### NO. 2.—AFFABILITY; URBANITY; TONE.

"A gentleman of excellent breeding and admirable discourse."—SHAKSPEARE.

FULLER says, that "William, Earl of Nassau, won a subject from the King of Spain, every time he put off his hat." More surely than the truth of this assertion, would be the truth of the assertion, that a dentist wins a patient every time he puts off a courteous act. The maxim of courts is, that manner is power; and we see the fact illustrated on all hands, however subtly. No one seriously claims exemption from the influence. An affable, urbane, high-toned appeal wins us, even though the cause be doubtful. Thus do manners oft-times prove a fortune, and may well be considered prominent among the elements of success with the dentist.

But how to acquire them?—how to direct our efforts? These are reasonable questions. First, then, let the man fully imbued with the importance of this *finish* to his character, next feel that "there is no excellence without great labor." Let him see the importance of thoroughness, deep-seatedness,—being assured that only as he approximates the *genuine*, is he secure; and that nothing but royal blood possessed will in any way shorten the road. Let him know that by his fruits he shall be known,—and how correctly!

"Beware you don't laugh," said a wise mother, "for then you show all your faults." But this mother referred to the *teeth*, and spoke when they were more plenty than now. Yet I tell you, a man no less shows his faults in these days, in opening his mouth, though he shows no teeth; so also does he in the more subtle language of his body. For weal or for woe, does his every word, his every act, tell on him. Thus significant are manners.

We may offer palliation for ill discourse, and not believe in pathognomy; but we yield our homage to the polished speaker, and our admiration to the elegant demeanor, and nine cases out of ten our suffrage accordingly, even at the protest of reserved merit.

Thus inseparable are the man and the manner; and thus important the thoroughness spoken of. For the manner can only be healthy when it proceeds from the character; or the character—man—must be sound to produee healthy manners.

Fortunately for us, we can mould our own character, and if our Adamic blood has run low, we have but to add culture, till in the end our factitious, and a more native manner, are indistinguishable; and certainly the former is as commendable as the latter.

How desirable is this depth and purity of manners!—this culture, education, and elevation of our natures, by thought, study, and the fine arts! How it redeems life! and makes friendship precious, as well as patience profitable!

Now if I can hope to have given a genuine impulse to the many vague desires which ever and anon seize us all, to be accomplished!—to ascend higher the elysian ladder of attainments, and enjoy the increased emoluments thereof; and have disabused the mind of the indolent or timid student or practitioner of the idea that nature gives for naught, or will tolerate a "wicked and slothful servant," or superficiality; and have shown that "every man is the architect of his own fortune,"—can carry this culture of manners to whatever point of success he chooses—I say if sentiments akin to these may have been produced already by my reflections, I will be excused from a more specific consideration of the triple heading above—Affability, polished discourse; urbanity, polished demeanor; and tone, serving to brace, and give effect and dignity to it all—and trust the careful student to appreciate the importance of the parts, in his study of a subject so fraught with pleasure and profit as a whole.

PIQUA, Ohio, *June*, 1862.

